# Utility of flexible interventional endoscopy in endoscopic sinus surgery: a case series

**DOI:** 10.1007/s00405-022-07655-6

**Published:** 2022-09-22

**Authors:** Yann Litzistorf, François Gorostidi, Karma Lambercy, Antoine Reinhard

**Affiliations:** Departement of Otorhinolaryngology Head and Neck Surgery, Centre Hospitalier Universitaire Vaudois (CHUV), University of Lausanne (UNIL), Lausanne, Switzerland

**Keywords:** Flexible endoscopy, Endoscopic sinus surgery, Draf III, Lateral recess

## Abstract

**Purpose:**

During endoscopic sinus surgery (ESS), difficult-to-reach pathologies need an extended endoscopic approach or an external approach. We started to use a flexible interventional endoscope (FIE) to evaluate the necessity of those approaches. The study's objective is to describe our experience and define patients who could benefit from this technique.

**Methods:**

We reviewed every patient who benefited from FIE associated with ESS at our tertiary University Hospital between January 2021 and February 2022.

**Results:**

During this period, we did 107 ESS, and 14 patients benefited from the FIE, representing 13% of our ESS. The median duration of the flexible endoscopy time was 14 min (4–38 min). We identified three groups of patients who can benefit from the FIE. The first one is for patients with a fungal infection, to control and to clean lateral recesses in a noninvasive manner. The second one is for patients with a pathology of the lateral frontal sinus, to remove the frontoethmoidal cells or mucocele with the biopsy forceps through the working channel. The third group is for patients with inverted papillomas, to precisely identify the insertion and to decide on the most appropriate surgical approach.

**Conclusions:**

In selected cases, using flexible endoscopy during ESS helps decide the optimal surgical approach and sometimes treat the pathology through a limited approach. Prospective studies for each group of patients are needed to confirm the benefit of this new combined procedure.

## Introduction

Endoscopic sinus surgery (ESS) uses rod endoscopes and rigid instruments to treat a wide range of sinonasal pathologies. Over the past years, it has become the gold-standard treatment for most cases, with good surgical and functional outcomes. However, pathologies located in some anatomical subsites remain difficult or impossible to reach, even with angled rod endoscopes and angled instruments. In these cases, a safe and complete treatment requires an extended endoscopic approach (EEA) or an external approach (EA), both associated with specific morbidities.

In the frontal sinuses, a Draf III approach ensures broad access to the sinus and efficient drainage of the cavity after surgery. Nevertheless, the Draf III procedure is associated with tedious postoperative care and a significant risk of secondary stenosis within 2 years [1–3]. Pathologies located in the lateral recess of the frontal sinuses are best accessed through an EA such as a supraorbital or a bicoronal approach. Both are associated with visible scarring, frontal paresthesia, significant blood loss, a risk for the orbital structures, and a small risk of cerebrospinal fluid (CSF) leakage [4].

Pathologies of the anterior wall of the maxillary sinus can be addressed through a Caldwell Luc approach which is associated with an incidence of persistent pain and hypoesthesia of the upper lip and teeth in up to 37% of cases [5]. Alternatively, the prelacrimal approach allows excellent control of the anterior maxillary wall with fewer risks for the anterior dentoalveolar nerve. However, in up to 31.5% of cases, the prelacrimal window is smaller than 3 mm, and the mobilization of the lacrimal tract can damage the lacrimal pathway resulting in persistent epiphora [6, 7].

In the sphenoid sinuses, the lateral recesses can be accessed through a trans-pterygoid approach associated with risks of lesions to the internal maxillary artery, the sphenopalatine ganglion, and the Vidian nerve, and the infraorbital nerve (trigeminal V2). In the literature, this approach is associated with a 10% risk of xerophthalmia and nasal dryness and a 12.8% risk of lesion of the infraorbital nerve with resulting facial hypo/anesthesia [8]. Variations with partial preservation of the pterygopalatine fossa have been proposed to lower these morbidities [9].

To date, the indications for EEA and EA depend on the pathology to be treated and on the patient's anatomy. Given the high variability of sinus anatomy, precise guidelines are lacking, and surgeons rely on their experience to choose the least invasive approach providing sufficient control. Improving the visualization and the surgical access to difficult anatomical subsites could lower the need for EEA and EA, thus the incidence of their complications, without compromising surgical control. To this end, we are now combining classical ESS techniques with flexible interventional endoscopy (FIE). In 2020, Png et al. first described using the flexible bronchoscope to treat a lateral frontal mucocele [10]. Lately, another group described the treatment of lateral frontoethmoidal cells obstructing the frontal sinus drainage pathway and the management of a fungus ball localized in the lateral recess of the frontal sinus using a FIE through a limited approach (Draf IIa) [11].

After realizing the advantages of this combined technique, we used it systematically in cases, where an EEA or an EA was planned. The primary endpoint was to evaluate if this combined technique could efficiently treat difficult-to-reach sinus pathologies and if some EEA or EA could be avoided. We describe our early experience and discuss the next steps of development.

## Materials and methods

**Fig. 1:**
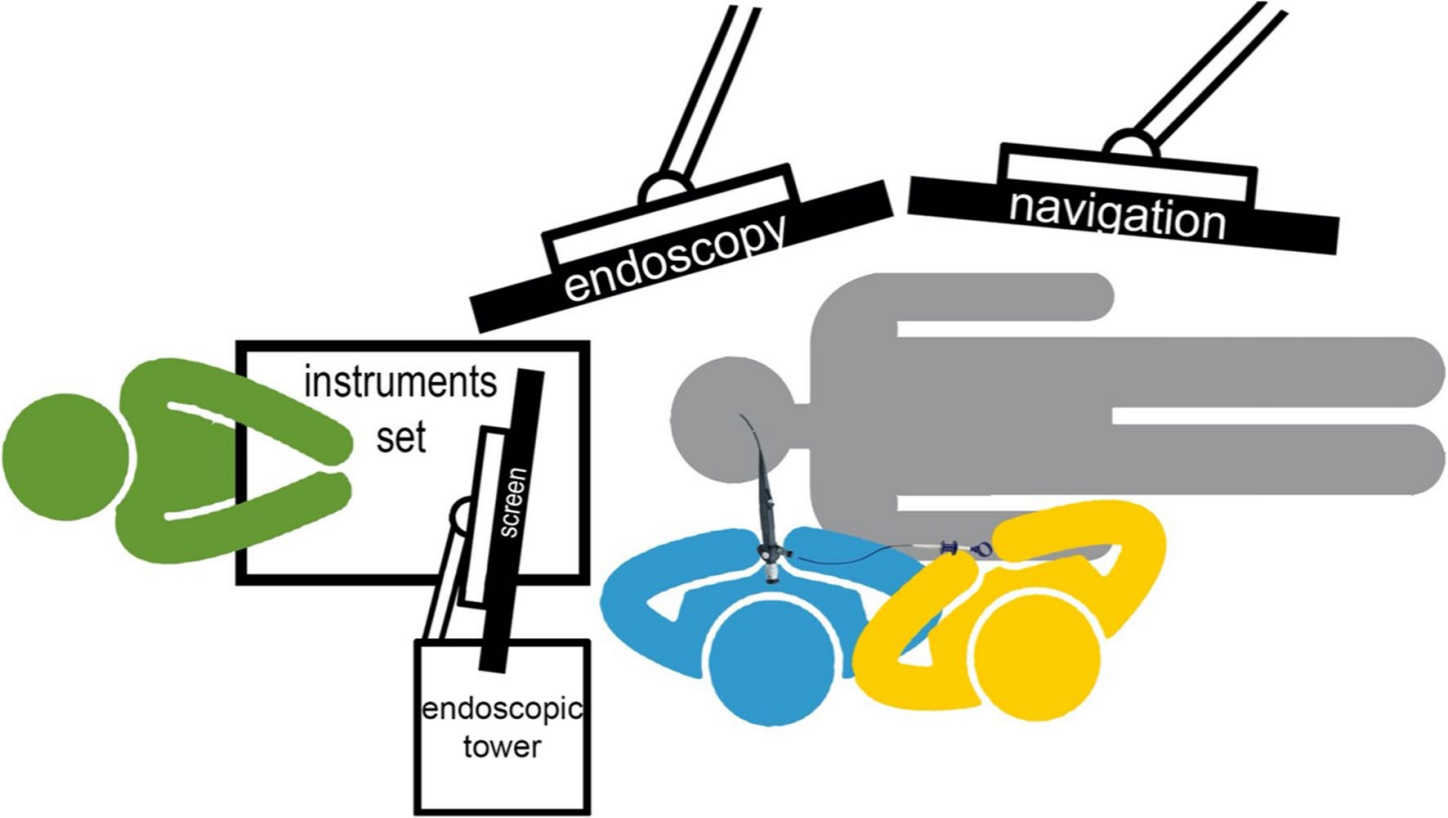
Layout of the patient, the equipment, and the personnel in the operating room during the flexible interventional endoscopy. In blue, the first surgeon is guiding the endoscope within the sinus cavities through the limited approach. In yellow, the second surgeon operating the flexible instrument or rinsing the camera through the working channel

After approval by the local ethics committee (CER-VD REQ-2022–00200), the medical files and image/video documentation of all patients treated with the combined ESS–FIE technique in our tertiary University Hospital (CHUV) between January 2021 and February 2022 were retrospectively reviewed. Patients’ data, surgical data, and follow-up information were retrieved for analysis. All surgeries were performed by two experienced surgeons, with neuronavigation control (Medtronic stealth station S8) and Hopkins’s rod telescopes (Karl Storz and Co), angled view of 0°, 45°, 70° coupled with a high-definition camera (Karl Storz and Co, IMAGE1™ H3-Z) and rigid angled instruments. The frontal sinus was accessed through a Draf IIa, the maxillary sinus through a middle meatal antrostomy, and the sphenoid sinus through a sphenoidotomy. The FIE was done with a four-hands technique by two experienced surgeons, one guiding the endoscope within the sinus cavities through the limited approach and the other operating a flexible instrument or rinsing the camera through the working channel (Fig. 1). In some cases, the FIE was used to visualize a particular recess while using a rigid angled instrument to open some cells or mucoceles safely under vision. The combined technique (ESS–FIE) was used in all cases, where the preoperative workup or intraoperative findings indicated that an EEA or an EA was likely. This technique was used through EEA when it had already been performed for a previous surgery. ESS–FIE was not used for malignant cases which were directly addressed through EEA or EA when required.

Two types of flexible interventional endoscopes were used. The Olympus BF-P190 bronchoscope (external diameter: 4.2 mm, working channel diameter: 2.0 mm, vision angle: 110°, angulation of 210° upward with no instrument and 180° with an instrument in the working channel, and 130° downward). It also has a rotation function of 120° to the right and left, which reduces the rotation of the operator's arm when working on the anterior wall of the maxillary sinus and the lateral part of the frontal sinus. The second endoscope was a sterile single-use bronchoscope, model aScope™ 4 broncho regular made by *Ambu*^®^ (external diameter: 5.0 mm, working channel diameter: 2.2 mm, vision angle: 85°, angulation up and down until 180° without an instrument in the working channel).

With both endoscopes, a syringe can be adapted to the working channel to clean the “chip-on-tip camera, to rinse difficult-to-access anatomical subsites, and to aspirate selective samples for microbiology analysis. A single-use standard oval biopsy forceps with a 5.0 mm cup opening with a 115 cm working length (Olympus FB-231D.A) was inserted through the working channel for selective mucosal biopsy sampling and to open thin osseous cells and mucoceles.

## Results

From January 2021 to February 2022, 107 ESS were performed in our institution, and 14 patients benefited from the combined ESS–FIE technique representing 13% of ESS. The median age of patients was 61 (IQR 19, range 17–84). The median duration of the procedures was 161 min (IQR: 46, range 85–216 min) with a median flexible endoscopic time of 14 min (IQR: 11, range 4–38 min), which represented 8% of the total operative time. All cases, grouped by pathologies, are shown in Table 1.

**Table 1:** Cases summary, grouped by pathologies

Case	Age	Sex	Follow-up (m)	Location	Pathology	Intervention with the flexible endoscope	Operation time (min): total/flexible e./%	Complication	Benefit
1	70	M	9	Sphenoidal	Fungus ball	Control and cleaning of the lateral recess	85/16/19	No	Comprehensive controle and treatment
2	77	M	7	Sphenoidal	Fungus ball	Control and cleaning of the lateral recess	103/5/5	No	Comprehensive controle and treatment
3	17	F	3	Maxillary	Fungus ball	Control and cleaning of the prelacrimal recess	171/15/9	No	Comprehensive controle and treatment
4	61	M	2	Maxillary	Fungus ball	Control and cleaning of the prelacrimal recess	156/17/11	No	Comprehensive controle and treatment
5	65	M	13	Frontal	Fungus ball/frontoethmoidal cell	Opening a frontoethmoidal cell through a Draf IIa	152/18/12	No	Avoid a Draf III
6	58	M	15	Frontal	Frontoethmoidal cell	Opening a frontoethmoidal cell through a Draf IIa	93/21/23	No	Avoid a Draf III
7	45	M	5	Frontal	Frontoethmoidal cell	Opening a frontoethmoidal cell through a Draf IIa	217/24/11	No	Avoid a Draf III
8	61	M	8	Frontal	Mucocele	Opening the lateral mucocele through a Draf IIa	216/23/11	Revision for a Draf IIb	Avoid an external approach
9	84	F	5	Frontal	Mucocele	Opening the lateral mucocele through a Draf IIa	189/13/7	No	Avoid an external approach
10	60	M	10	Frontal	Inverted papilloma	Identification of the site of origin	182/5/3	No	Indication of the bi-coronal approach
11	28	M	9	Maxillary	Inverted papilloma	Identification of the site of origin	176/10/6	No	Indication of the prelacrimal approach
12	45	M	5	Maxillary	Inverted papilloma	Identification of the site of origin	161/10/6	No	Indication of the prelacrimal approach
13	68	M	5	Maxillary	Inverted papilloma	Identification of the site of origin	162/6/4	No	Indication of the prelacrimal approach
14	66	F	2	Maxillary	Inverted papilloma	Identification of the site of origin	129/4/3	No	Avoid the prelacrimal approach

**Fig. 2:**
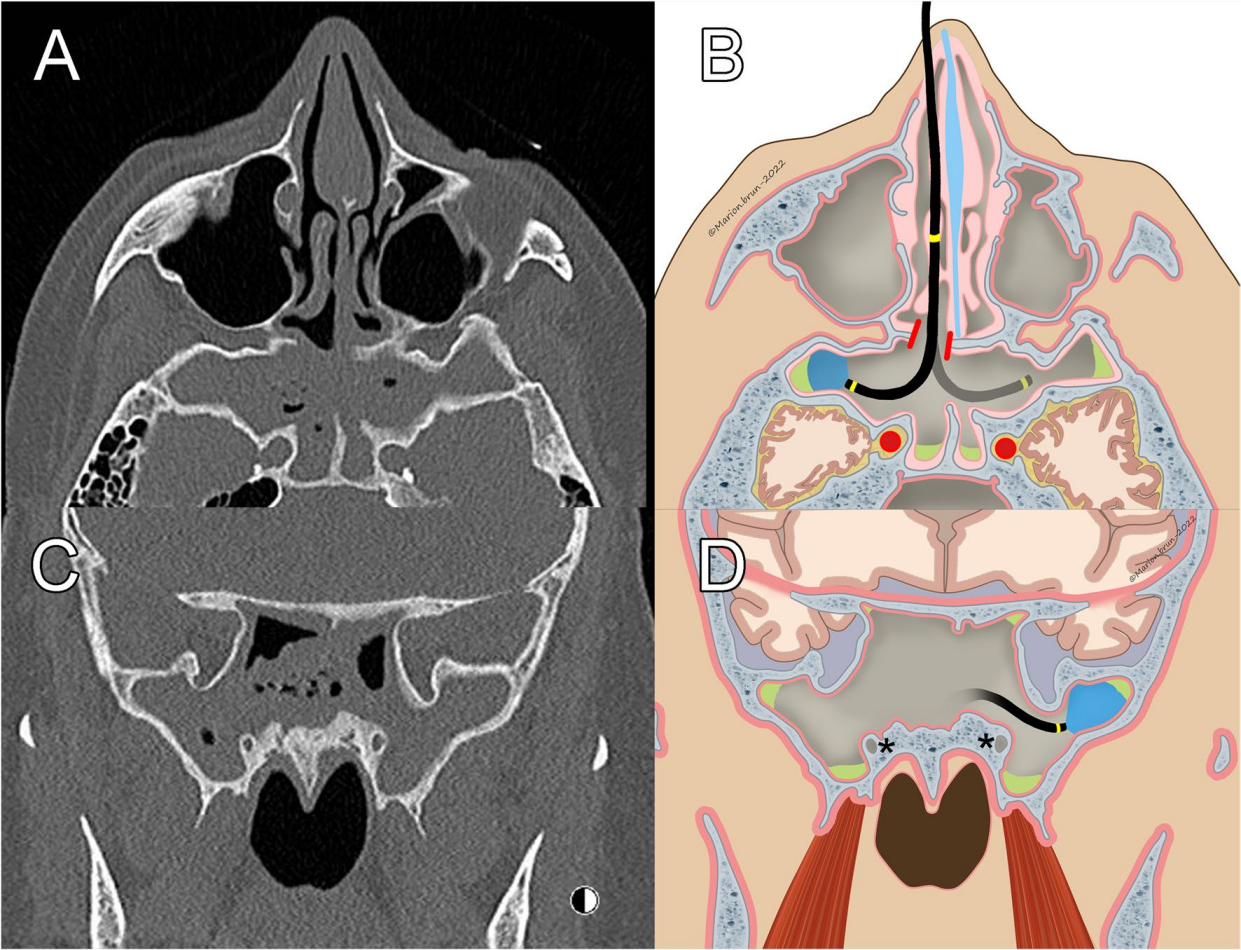
Illustration of case number 1. **A** Axial view of the CT scan showing the bilateral sphenoidal sinusitis with large lateral recesses and typical bone thickening. **B** Schema of the flexible endoscopy accessing the lateral recess through a sphenoidotomy. **C** Coronal view of the CT scan showing the lateral recess of the sphenoid. **D** Schema of the flexible endoscopy accessing the lateral recess. The Vidian nerve is well visualized and symbolized by the *. The residual fungus material is symbolized in green

Five patients had fungal sinusitis, 2 of which were maxillary, 2 sphenoidal, and one frontal. In all of them, after completion of the traditional ESS procedures, FIE allowed the observation and the complete removal of residual fungal material despite prior meticulous rinsing and cleaning with rigid instruments. None eventually required an EEA, and none relapsed during follow-up (median 7 months, IQR 6, range 2–13). Figure 2 illustrates case number 1, in which FIE was used for 16 min to clean and to control the recesses through a sphenoidotomy. This case represents the added value of FIE in the management of difficult-to-reach fungal sinusitis. Indeed, without FIE, such a case would have required an enlarged sphenoidotomy with higher bone exposure and potential risk of Vidian nerve damage.

**Fig. 3:**
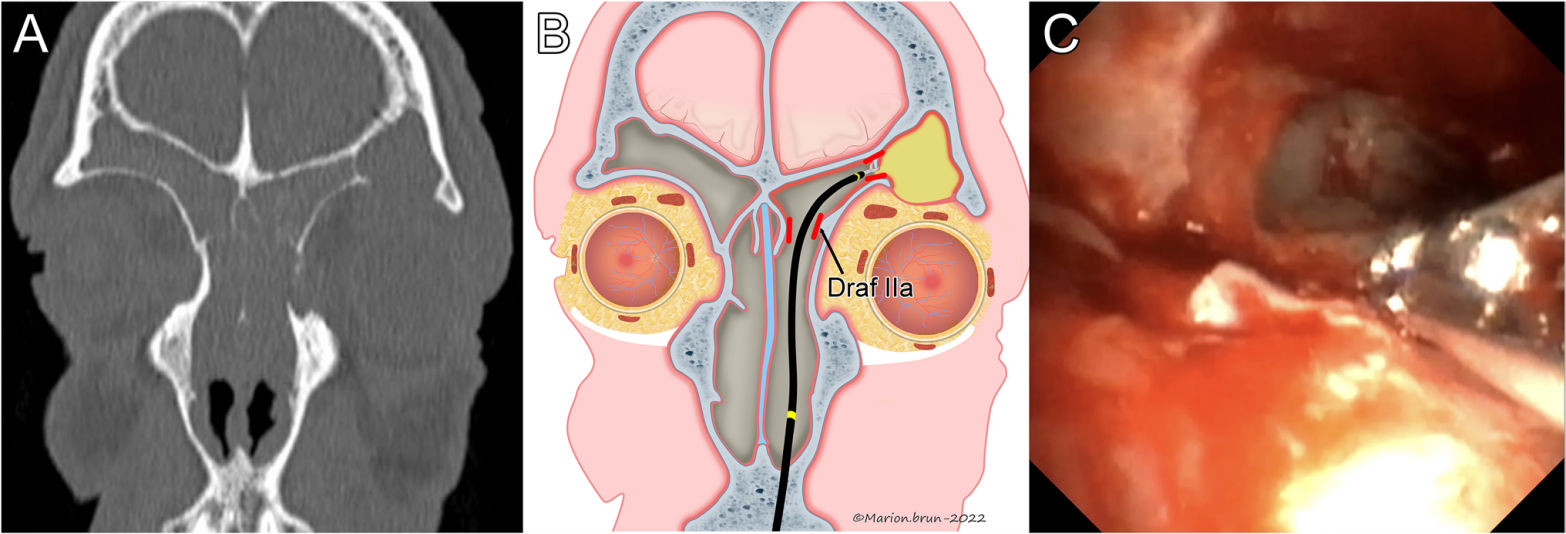
Illustration of case number 8. **A** Coronal view of the CT scan showing the frontal lateral symptomatic mucocele with a lysis of the orbital roof. **B** Schema showing the endoscopic removal the mucocele’s wall through a Draf IIa with the biopsy forceps. **C** View with bronchoscope showing the remaining upper wall of the mucocele and the biopsy forceps through the working channel that will grab this wall. On this picture, the mucocele has already been rinsed

Five patients had frontal sinus drainage obstruction, 3 of which had lateral frontoethmoidal cells, and 2 had a lateral mucocele. All five cases were addressed through a classical Draf IIa approach, and the residual cells/mucoceles were successfully removed with the flexible biopsy forceps used through the working channel of the FIE. Thus, this technique initially prevented the need for an EEA or EA in all five cases. The median FIE operating time was 21 min (IQR: 6, range 5–38). We observed one patient with a recurrence of symptoms during the median follow-up of 8 months (IQR: 5, range 5–15). This patient corresponds to patient 8 in Table 1 and is illustrated in Fig. 3. He was known for aspirin-exacerbated respiratory disease (AERD) and presented with a lateral symptomatic frontorbital mucocele of the left frontal sinus. 5 months after initial surgery, he presented with a new palpebral swelling. The CT scan showed an open and aerated mucocele, but the nasofrontal canal was blocked due to recurrent polyps. As illustrated in Fig. 3A, the roof of the orbit was eroded, and the patient experienced symptoms at the slightest increase in pressure in the frontal sinus. He required revision surgery to convert the Draf IIa into a Draf IIb. He had a favorable evolution in combination with a biological treatment (dupilumab). In this case, the ESS–FIE could avoid an external approach but not an extended endoscopic approach perhaps because the underlying disease was not well enough controlled. This is our only patient who needed revision surgery.

**Fig. 4:**
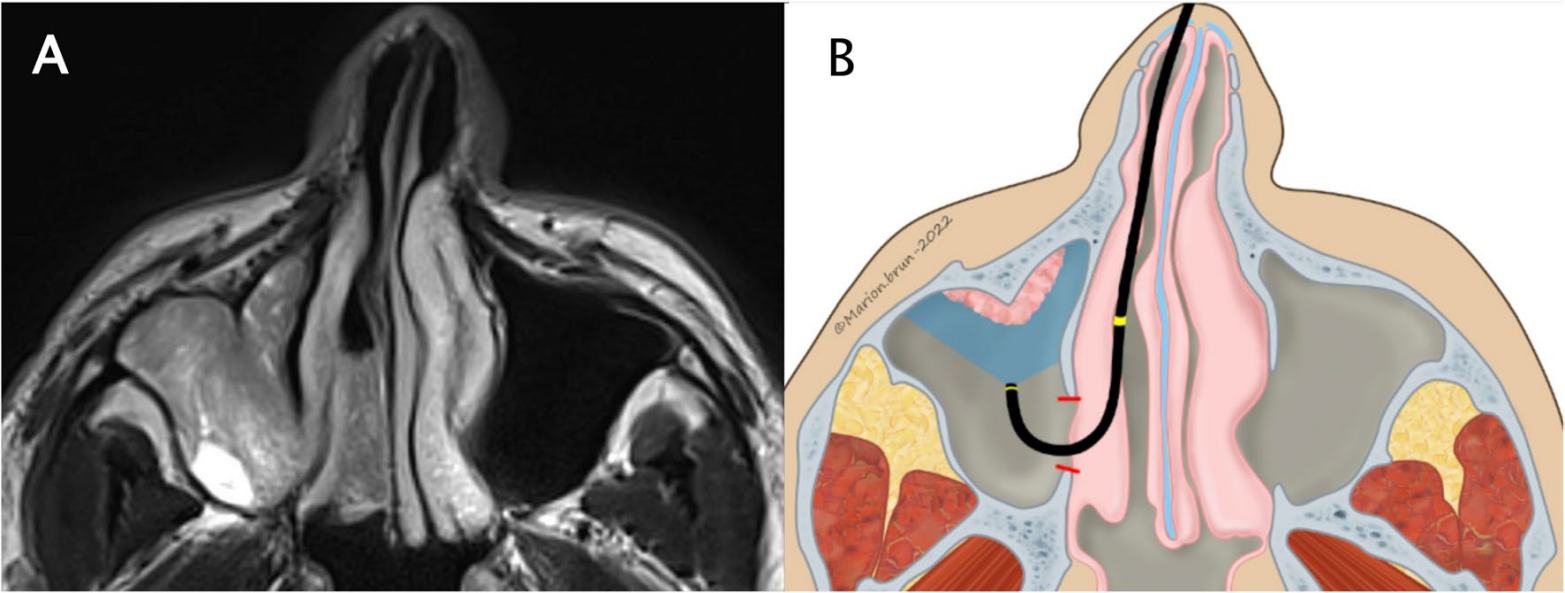
Illustration of case number 12. **A** MRI: axial view passing inferiorly of the lacrimal duct, showing the insertion of the inverted papilloma on the anterior wall of the maxillary sinus. The lacrimal recess is filled with cerebriform tissue, but the insertion looked more lateral. **B** Schema of the flexible endoscopic showing the true extension to the lacrimal recess

**Fig. 5:**
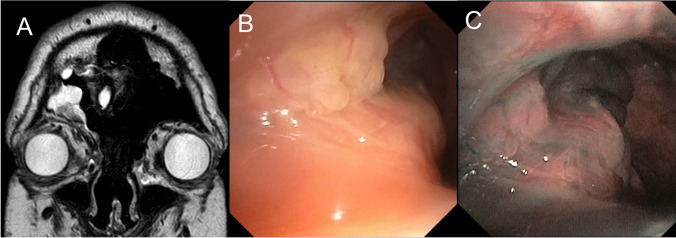
Illustration of case number 10. **A** MRI: coronal view showing the recurrence of the inverted papilloma on the lateral recess of the right frontal sinus. **B** Endoscopic view with the white light. **C** Endoscopic view with the Narrow-Band Imaging enhancing the vascular pattern

Five patients had inverted papilloma (IP), four in the maxillary sinus, and one in the frontal region. For all of them, the location of the IP implantation was unclear on preoperative CT and MRI thus combined ESS–FIE was planned to select the best definitive approach. In all five patients, FIE with narrow-band imaging allowed the precise identification of the IP implantation through a limited endoscopic approach. The most appropriate EEA or EA was then selected to remove the IP completely with a selective cauterization and/or drilling of the implantation. In our 4 cases of IP located on the lacrimal recess or the anterior wall of the maxillary sinus, a careful examination of the mucosa with the FIE showed an extension of the disease that was not accessible through meatotomy in 3 cases. Thus, we made three prelacrimal approaches, and we could safely avoid one prelacrimal approach. Figure 4 shows the example of patient 12, who had an IP coming from the anterior wall of the maxillary sinus. MRI showed that the lacrimal recess was filled with cerebriform tissue, but it was not possible to determine whether it was the implantation site or the exophytic part of the IP. With the 70° rod endoscope, the lacrimal recess could not be visualized. FIE allowed the complete exploration of the mucosa of the maxillary sinus and, in this case, to confirm the indication of the prelacrimal approach to remove the mucosa of the prelacrimal area. Figure 5 shows the case of patient 10, who presented the 4th recurrence of inverted papilloma in the frontorbital groove and had previously undergone a Draf III. The FIE associated with the Narrow Band Imaging (NBI) allowed to identify a diffuse recurrence with a large and multifocal insertion which was not visible on the MRI. Therefore, a bicoronal approach was selected to treat the recurrence optimally.

We had no immediate and short-term complications related to the use of the FIE with a median follow-up time of 6 months (IQR: 4, range 2–15). We could avoid an EEA in 6 out of 14 interventions and, therefore, reduce the surgeries' morbidity.

## Discussion

Minimizing the use of extended endoscopic approaches (EEA) and external approaches (EA) is essential to decrease the incidence of their intrinsic complications and morbidities. However, it is crucial not to jeopardize the surgical control of the diseases. In our hands, in selected cases, the combination of classical endoscopic sinus surgery (ESS) techniques with flexible interventional endoscopes (FIE) is a valuable tool to efficiently treat difficult-to-reach pathologies through limited endoscopic approaches and for intraoperative decision-making when selecting the most appropriate EEA or EA.

In this retrospective series, the selection of patients was based on the pathologies, the preoperative imaging, the intraoperative findings, and the surgeons’ experience. It is, therefore, difficult to generalize our findings to other cohorts. However, our early experience helped define indications and contra-indications. We identified three types of cases that benefit from this combined procedure. These are patients with difficult-to-reach infectious diseases, benign neoplasia, and some obstruction of the frontal sinus drainage pathway by lateral frontoethmoidal cells or by mucoceles. Typical difficult-to-reach locations that can be reached with the FIE include the anterior wall and lacrimal recess of the maxillary sinuses, the lateral recesses, the supraorbital region of the frontal sinuses, and the lateral recesses of the sphenoid sinuses.

In cases of fungus ball, FIE allowed a comprehensive control of residual fungal material in every recess after standard ESS and a treatment of those by selectively rinsing under vision. We used FIE only when the sinuses were not fully controllable with angled rod endoscopes. This was the case for 5 patients out of 22 (22.7%) patients treated for fungus infection during the study period. We found fungal residue in every patient, meaning that without FIE we would have had at least 22.7% of patients with residue during the post-operative follow-up, or those patients would have needed an EEA to treat completely the fungal material. This finding is consistent with the current literature stating that 21% (18/86) have residual fungus at the time of the first postoperative rinse [12] and that 5% of mycotic sinusitis required revision surgery [13, 14]. Based on our experience, we believe that this technique could decrease the need for EEA while preventing recurrences. This needs to be confirmed in a larger cohort study with a long follow-up.

From our experience, one other clear advantage of the combined ESS–FIE technique is the enhanced visualization of lateral recesses and the possibility for selective tissue sampling.

Obstruction of the frontal sinus drainage pathway can be difficult to address through minimally invasive approaches in ESS making EEA sometimes necessary. Draf IIa procedure induces less mucosal trauma and less bone exposure leading to quicker post-operative healing and less pain [3]. Draff III is associated with more postoperative follow-up visits and a risk of delayed restenosis. This risk is increased in case of allergic fungal sinusitis, recurrent staphylococcus aureus infection[15], and when neoosteogenesis is already observed on preoperative CT [16]. For those reasons, we tried to be as little invasive as possible for primary cases. Here, we describe the cases of five patients in which the combination of ESS with FIE allowed the complete removal of obstructive frontoethmoidal cells through a limited Draf IIa. As discussed above, one of the patients eventually needed a revision and a conversion of the Draf IIa to a Draf IIb followed by biological treatments. All other 4 patients were treated through a minimally invasive approach without recurrence at the last follow-up. In this subgroup of patients, the median FIE operative time was higher, because the osseous septae of obstructive frontoethmoidal cells had to be removed pieces after pieces with the flexible biopsy forceps passed through the working channel. The limitations occurred when the bony septa cannot be grabbed, because it is too thick or too flat. We overcame this limitation by opening the cell with a rigid angled instrument under the vision of the flexible endoscope and then grabbed bony septa with the biopsy forceps. When considering the indication for a minimally invasive approach, it is important to consider the activity of the underlying inflammatory disease and the aforementioned risk for the recurrence of nasofrontal duct stenosis. Despite positive initial experience in these five cases, the benefit of combined ESS–FIE on the symptom-free interval, the rate of delayed restenosis, and revision surgery need to be confirmed in larger, longitudinal cohort studies.

In cases of sinonasal tumors, the principle of surgery is to remove the bulk of the tumor and, most importantly to completely excise the site of implantation. Preoperative imaging (CT and MRI) is often sufficient to determine the site of origin and the most appropriate EEA or EA is chosen accordingly. However, it is sometimes impossible to reliably determine the exact implantation of a tumor on the preoperative imaging, which makes the choice of the approach difficult. In some cases, it is retrospectively evident that a less invasive approach would have been sufficient. We describe here five cases of inverted papilloma (IP) in which the use of FIE was very useful to precisely determine the site of implantation, leading to a confident intraoperative choice of the most appropriate approach. The definitive benefits of this tool will need to be tested in larger comparative studies.

With this limited experience of 14 cases, we did not face any technical problems or complications linked to the use of FIE. Repeated rinsing of the camera head ensures a clear vision and a safe navigation in the critical sinus anatomy. Procedures were performed confidently without fear of damaging the skull base or the orbit. However, the safety of this technique will need to be confirmed in a larger cohort.

As with any new surgical technique, there is a learning curve to master the use of a bronchoscope within the sinonasal cavities, but most otolaryngologists are familiar with nasofibroscopes and sometimes flexible bronchoscopes which facilitates the learning. In our hands, one clear limitation of the technique is the absence of tactile feedback with the FIE and the impossibility to apply significative force to the flexible biopsy forceps (as compared to what is possible with rigid, angled instruments), because the FIE moves backward when more force is applied to the flexible instruments used through the working channel. This limits the usefulness of FIE to excise harder bone septa or osteomas for instance. We are now exploring the development of through-the-scope burr and FIE anchoring systems to overpass this limitation.

## Conclusions

The combination of ESS with FIE, in selected cases, provides additional surgical control without the need for more invasive approaches. We identify three main indications, where FIE is of benefit. The first one is for the obstruction of the frontal sinus drainage pathway by fronto-ethmoidal cells/mucocele which can be treated through a Draf IIa approach. The second one is for the fungal sinusitis localized in difficult-to-reach subsites, which can be reliably visualized and flushed with the FIE. The third one is for selected benign sinonasal tumors to help determine the origin of the tumor and thus help to decide the best endoscopic approach. Thanks to the development of new instruments dedicated to flexible interventional endoscopy, this combined technique reveals new perspectives toward less invasive, better-tolerated surgeries.
